# Mechanical Property, Efficacy, and User Experience of an Innovative Wearable Device in Preventing Fall-Induced Injuries

**DOI:** 10.1093/geroni/igae066

**Published:** 2024-07-12

**Authors:** Kaixin Hu, Zhongyang Guan, Zhijie He, Siran Luo, Hongbin Fang, Yan Zhang, Li Ding, Ying Xu, Liuya Jiang, Conghui Fu, Xiaoqing Zhao, Jie Jia, Chenkai Wu

**Affiliations:** Department of Rehabilitation Medicine, Huashan Hospital, Fudan University, Shanghai, China; Global Health Research Center, Duke Kunshan University, Kunshan, Jiangsu, China; School of Population Health, Faculty of Health Science, Curtin University, Bentley, Western Australia, Australia; Department of Rehabilitation Medicine, Huashan Hospital, Fudan University, Shanghai, China; Global Health Research Center, Duke Kunshan University, Kunshan, Jiangsu, China; Institute of AI and Robotics, Fudan University, Shanghai, China; Suzhou Youxing Health Technology Co., Ltd, Suzhou, Jiangsu, China; Department of Rehabilitation Medicine, Huashan Hospital, Fudan University, Shanghai, China; Shanghai Jinshan Zhongren Aged Care Hospital, Shanghai, China; Department of Rehabilitation Medicine, Shanghai Jing’an District Central Hospital, Shanghai, China; Shanghai Jinshan Zhongren Aged Care Hospital, Shanghai, China; Suzhou Youxing Health Technology Co., Ltd, Suzhou, Jiangsu, China; Department of Rehabilitation Medicine, Huashan Hospital, Fudan University, Shanghai, China; National Clinical Research Center for Aging and Medicine, Huashan Hospital, Fudan University, Shanghai, China; Global Health Research Center, Duke Kunshan University, Kunshan, Jiangsu, China

**Keywords:** Falls, Hip fracture, Injury

## Abstract

**Background and Objectives:**

With the global population aging at an unprecedented pace, the imminent surge in falls and fall-induced injuries necessitates urgent attention. Innovative assistive technologies are crucial in addressing this daunting challenge. This study aimed to evaluate the mechanical properties, efficacy, safety, and user experience of the Intelligent Bone Protection Vest (IBPV), a novel, reusable, non-airbag wearable device.

**Research Design and Methods:**

The IBPV integrates a machine learning-based algorithm for real-time monitoring of wearer motion and a unique honeycomb-structured foldable cushion for fall impact attenuation. We evaluated the impact attenuation capabilities of the IBPV and conducted 2 human subject studies to assess its efficacy and safety. Additionally, semistructured interviews were conducted to qualitatively explore its usability, safety, and opportunities for enhancement.

**Results:**

The compression tests confirmed the energy absorption capacity of the honeycomb-structured foldable cushion. In over 800 fall tests involving 14 young and middle-aged subjects using a touchdown fall test, as well as 7 older subjects using a novel fall simulation test, the IBPV demonstrated an overall protection rate exceeding 84%.

**Discussion and Implications:**

These results underscored the potential of the IBPV in reducing fall-induced injuries by mitigating the impact force on the hip during falls. Future studies with more rigorous design are needed to confirm whether this active wearable device may serve as a dependable fall protection product.

**Translational Significance:** The Intelligent Bone Protection Vest (IBPV) integrates a machine learning-based algorithm to monitor wearers’ motion characteristics in real-time and a unique honeycomb-structured foldable cushion to attenuate the impact of falls. Unlike the mainstream active wearables, the IBPV does not utilize airbag technology. Our investigation emphasizes the potential of the IBPV in diminishing the occurrence of injuries resulting from falls, particularly by proficiently mitigating the impact force on the hip. This may offer fresh perspectives on optimizing and developing active wearable technology products for preventing fall-induced injuries in the elderly.

## Background and Objectives

Falls and the associated injuries represent a pressing global health concern. Annually, approximately 37.3 million severe falls that require medical attention occur worldwide, resulting in the loss of over 17 million disability-adjusted life-years (World Health Organization, 2021). These injuries can lead to prolonged healthcare usage and high costs (Shumway-Cook et al., 2009). The risk of falls increases with age; for instance, about one in four adults aged 65 and older in the United States reported at least one fall in 2020 (Peel, 2011). In addition to aging, multiple demographic characteristics, such as being female and single, as well as health conditions, including obesity and cognitive impairment, are significantly associated with a heightened risk of falling (Bergen et al., 2021; Mitchell et al., 2014).

Fall-induced injuries range from minor soft tissue damage to severe injuries, such as head trauma and fractures of the hip, spine, upper arm, forearm, pelvis, hand, and ankle (Rubenstein & Josephson, 2002; Stevens & Olson, 2000). Among these severe injuries, hip fractures—a devastating type of fracture due to their high risk of morbidity, mortality, and disability (Giversen, 2007; Johnell & Kanis, 2004; Kanis et al., 2012; Papadimitriou et al., 2017)—are considered the most common, accounting for 75% of fall-related hospitalizations (Bradley, 2013; Cummings & Melton, 2002). Furthermore, experiencing falls and the resulting injuries, or even the fear of potential consequences, such as social isolation, loss of independence, decreased self-confidence, and the possibility of needing long-term care facility admission, could lead to severe depression and anxiety (Hull et al., 2013; Scaf-Klomp et al., 2003). This underscores the urgent demand for innovative assistive technologies and devices capable of preventing or mitigating fall-induced injuries, particularly those involving the hip.

In recent years, considerable endeavors have been devoted to developing and evaluating technologies, such as wearable devices, aimed at reducing peak impact forces on the hip during falls, thereby potentially diminishing the risk of fall-induced injury. A narrative review by Tarbert et al. (2023) identified two primary categories of wearable devices that have been designed for this purpose: passive and active protection mechanisms. Passive wearable devices, exemplified by hip protectors, typically feature a convex shape and are secured to the hip through specialized clothing. These hip protectors, integrating soft or hard padding to reduce the impact force sustained during a fall, a rigid shell to divert fall energy away from the hip, or a combination of these features (Tarbert et al., 2023), have been advocated as a cost-effective and relatively immediate approach for prevention within the long-term care environment (Korall et al., 2015). Although these products require minimal training for usage, they confront challenges related to user adherence, primarily stemming from self-reported discomfort attributed to the utilization of cumbersome devices, the manifestation of associated side effects such as skin irritation and urinary incontinence, as well as the common need for assistance when donning and doffing the devices, particularly among older adults. Moreover, users are disinclined to wear these protectors in the bath or shower, where many falls occur (Tarbert et al., 2023).

In contrast, active protection devices, often featuring airbag technology, maintain continuous surveillance of the wearer’s body movements. Upon detection of a fall by integrated sensors, these devices rapidly inflate the airbags before the wearer’s hip contacts the ground, thereby effectively mitigating the impact force. Recent advancements in this field have given rise to sophisticated smart airbags, such as the Tango Belt, which harness machine learning algorithms for fall detection (Quigley et al., 2019; Tarbert et al., 2023). In instances of severe hip-impacting falls, the Tango Belt deploys automotive-grade airbags that envelop both hips when the wearer contacts the ground (Quigley et al., 2019). It can communicate mobility data, urgent fall alerts, and notifications of assistance requirements (e.g., low battery status) to caregivers (Quigley et al., 2019). However, it is important to acknowledge that the cost of these active wearables exceeds that of passive wearable devices, and many of them utilize single-use airbags that become nonreusable once deployed unless undergoing refurbishment. This gives rise to several notable concerns, including (i) effectiveness: airbags may not deploy in all falls with the potential to cause hip injuries as manufacturers aim to reduce the false positive rates; (ii) affordability: the financial and time costs associated with refurbishing these devices, which could act as a barrier to widespread adoption; and (iii) usability: similar to passive wearables, these products are often inconvenient and uncomfortable to wear in specific environments, such as during bathing and showering, where many falls commonly transpire (Tarbert et al., 2023).

**Figure 1.**
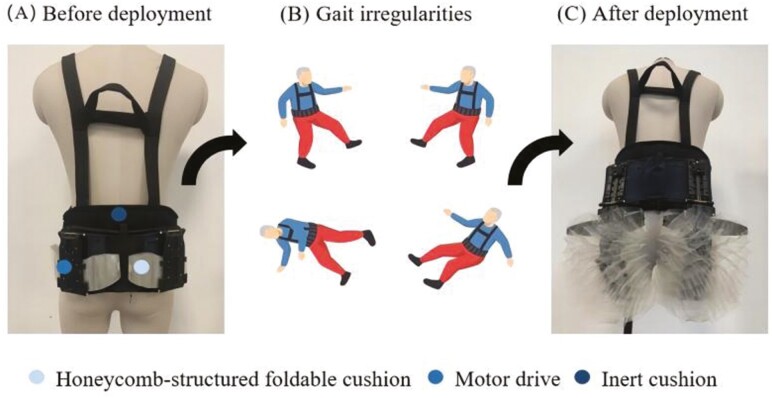
The intelligent bone protection vest.

The Intelligent Bone Protection Vest (IBPV; Figure 1) represents an active non-airbag wearable characterized by its non-reliance on airbag technology and its distinctive feature of equipment reusability, achieved through its innovative honeycomb-structure foldable cushion. This advanced wearable employs a machine learning-based algorithm to continuously monitor the real-time motion characteristics of the wearer. In the event of a fall, the IBPV deploys the honeycomb-structured foldable cushion to act as a buffer upon contacting the ground during a fall to reduce the risk of fall-induced injuries.

To date, numerous studies have evaluated the efficacy and compliance of hip protectors in preventing fall-induced injuries, particularly hip fractures, through randomized trials (Cameron et al., 2003; Chan et al., 2000; Kannus et al., 2000; O’Halloran et al., 2004). Furthermore, several investigations have explored the factors influencing the acceptance and compliance with hip protectors, utilizing qualitative methodologies such as semistructured interviews (Hall et al., 2019; Milisen et al., 2011). Building upon these methodologies, we comprehensively assessed the mechanical properties, effectiveness, safety, and user experience of the IBPV. The goal was threefold. First, we assessed the impact-attenuation ability of the honeycomb-structured foldable cushion. Second, we conducted two complementary human subject studies to identify the effectiveness and safety of the IBPV. The first study involved a touchdown fall test with young and middle-aged participants, whereas the second focused on a non-touchdown fall simulation test with older participants. Third, we conducted semistructured interviews to qualitatively evaluate the usability, safety, and opportunities for further optimization of the IBPV.

## Research Design and Methods

### Equipment

The IBPV comprises several key components, including an inertial measurement unit (IMU) motion sensor, a motor drive, a honeycomb-structured foldable cushion, and inert cushions (Figure 1). It collects accelerometer and gyroscope data at a frequency of 100 Hz via an on-board IMU chip. This generates six-dimensional data, with each sensor contributing *x*, *y*, and *z* dimensions. For the IBPV, predicting an impending fall is framed as a classification problem that utilizes IMU motion sensor readings from a specific time window to determine if the wearer will impact the floor within 300 ms after the time window ends. This 300-ms interval is designed to ensure that the honeycomb-structured cushion fully deploys by the time of impact. The motion sensor time window provides rich information about deviations in the wearer’s body position from regular movement in the early stage of a fall. Multiple features are derived from this time series and fed into a machine learning model running continuously on the device. To train the model, we collected sensor data from human subjects who simulated a wide variety of falls in a protected environment. The data were then annotated with the exact time of impact. Negative samples in the training data included sensor data collected while human subjects went about their normal activities, including actions similar to falling, such as sitting down and descending stairs. Upon identifying a fall, the sensor transmits signals to activate the motor drive, rapidly deploying the honeycomb-structured foldable cushion to cover and shield the wearer’s hip in 0.2 seconds, effectively reducing the impact force (Supplementary Table 1). Once deployed, both the motor drive and the honeycomb-structured cushion can be conveniently folded and reset for future use. In addition, the inclusion of inert cushions that envelop the wearer’s side hips and lumbar spine offers additional protection to these critical areas.

### Setting and Subjects

Two pilot studies were conducted between February 2023 and May 2023 at 4-m-long, 4-m-wide indoor test sites at Duke Kunshan University campus and Suzhou Youxing Health Technology. Study participants were recruited through social media, community outreach, and information shared by acquaintances. All potential participants were interviewed by telephone and were invited to the study sites if they: (i) were 18 to 75 years old, (ii) did not have a history of cardiovascular disease, and (iii) were at least 1.4-m tall and weighed less than 90 kg. Eligible participants were invited to the test sites, where engineers selected equipment of the appropriate size for each participant. As per engineers’ guidelines for fall simulations, all participants initially underwent the first fall test. Those who experienced discomfort during this initial test could withdraw from the subsequent fall tests. A total of 14 young and middle-aged adults (18–40 years) participated in the touchdown fall test. Fourteen older adults (62–73 years) participated in the non-touchdown fall simulation test, among which seven withdrew after the initial test; all participants conducted a semistructured interview. The study protocol was approved by the institutional review board of the Shanghai Jinshan Zhongren Aged Care Hospital (number: 202307). All the included subjects provided written informed consent for participation.

### Compression Experiments

**Figure 2.**
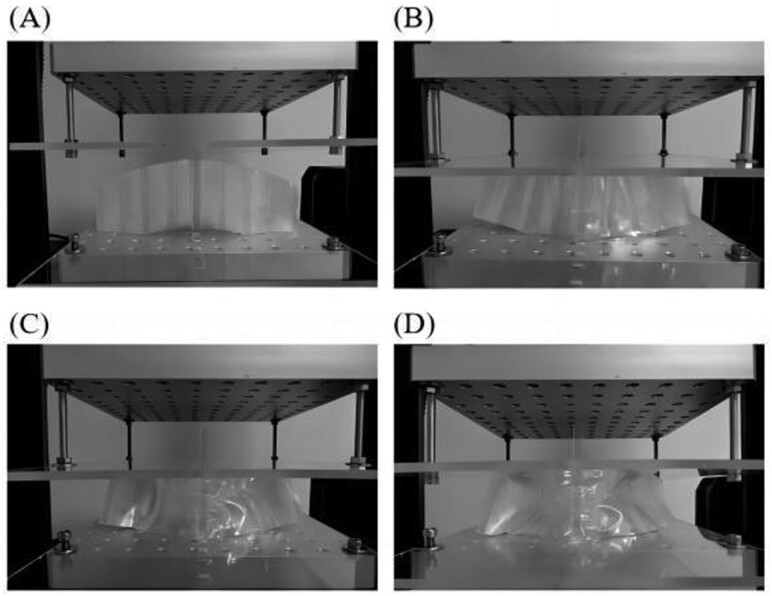
Sequential deformation process of the honeycomb-structured foldable cushion.

We performed compression experiments to analyze the impact-attenuation ability of the honeycomb-structured foldable cushion. The experimental setup and process for compression are shown in Figure 2. The experiments involved bi-axial quasi-static compression using a universal testing machine (5965, Instron). The lower platen remained stationary during compression, whereas the upper platen applied controlled displacement loading. The specimen was centered on the lower platen, and the force–displacement curves were continuously captured by measuring the supporting force and displacement of the upper platen up to a load of 1,000 N at a deformation rate of 10 mm/min. Each of the two tested samples underwent three repetitions to ensure reliability.

### Touchdown Fall Test

**Figure 3.**
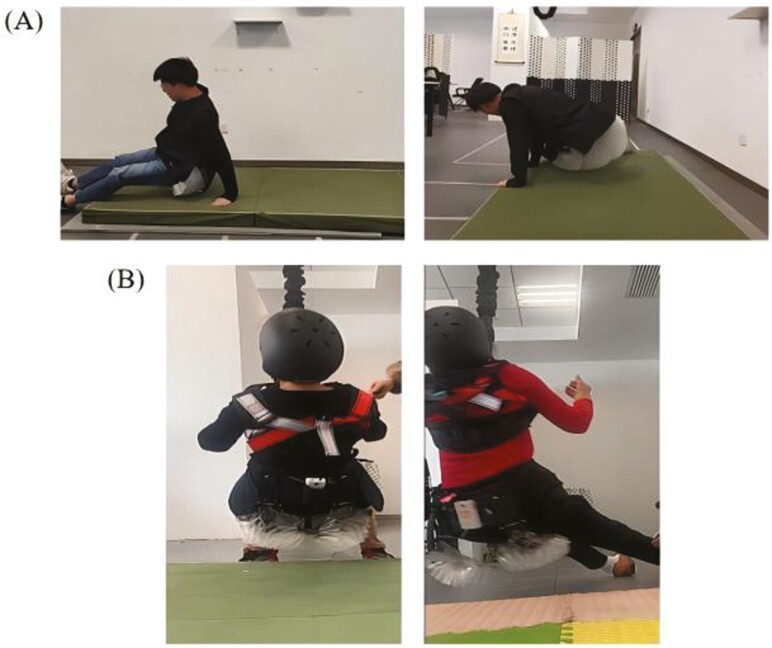
Fall tests images. (A) Touchdown fall test. (B) Non-touchdown fall simulation test.

During the touchdown fall simulation test, each participant was outfitted with the IBPV and a helmet with assistance from staff members. They were then instructed to execute falls onto gymnastic mats (Figure 3A). Participants were directed to mimic falling movements resembling natural falls in various real-life scenarios (e.g., falls in four directions: front, back, left, and right). Each simulated fall was replicated a minimum of five times.

### Non-Touchdown Fall Simulation Test

During the non-touchdown fall simulation test, each participant was equipped with the IBPV, a helmet, and an elastic suspension device with assistance from staff members. They were then asked to execute non-touchdown falls—their hips came close to but did not contact the ground (Figure 3B). All participants were asked to replicate falling movements resembling natural falls in various real-life scenarios. Each simulated falling motion was repeated a minimum of five times.

### Quantitative Data Collection

Demographic characteristics (age) and anthropometric data were gathered at study sites before the fall tests, including age, sex, height, weight, and body mass index (BMI) calculated as body weight in kilograms divided by standing height in meters squared. The falling process was captured on video, whereas the motion data produced during the falls were collected by the memory card within the IBPV.

### Qualitative Data Collection

Semistructured interviews were conducted among participants in the non-touchdown fall simulation test. During the interviews, subjects were invited to share their wearing experiences and provide insights into areas of improvement for the wearable device. The interview questions are listed in Supplementary Table 2.

### Analytic Approach for Fall Protection Results

The quality of each fall was assessed by engineers based on the recorded video to determine if it was realistic, natural, and met the criteria for fall data collection. The motion data collected by the memory card during each fall were paired with the video-recorded falling process. Based on the combined video and motion data, three categories of fall protection outcomes were identified: (i) successful protection: the honeycomb-structured foldable cushion effectively deployed and covered the subject’s hip before it made contact with the floor, (ii) failed protection: the honeycomb-structured foldable cushion either failed to deploy, or failed to cover the subject’s hip before it struck the floor, and (iii) invalid protection: the honeycomb-structured foldable cushion deployed due to exceptional circumstances, such as “false-positive” falls.

### Analytic Approach for Wearing Experience

The interviews were conducted and transcribed verbatim by one author, Y. Zhang, and subsequently, all transcriptions underwent thorough review and correction for accuracy by another author, S. Luo. For qualitative data analysis, an inductive content analysis approach was employed, including initial open coding, construction of categories, and abstraction into themes, was used to analyze the qualitative data (Patton, 2015). Open coding entailed multiple readings of the transcripts by two authors (Z. Guan and S. Luo) individually, with disagreement resolved by another author (C. Wu); headings and notes were written in the transcripts while reading them. Higher-order headings were then classified based on similarities and differences observed. Finally, the abstraction of categories was put into initial themes. These initial themes were then further discussed and compared among all authors, and a consensus was reached regarding the final themes and subthemes.

## Results

### Mechanical Properties

**Figure 4.**
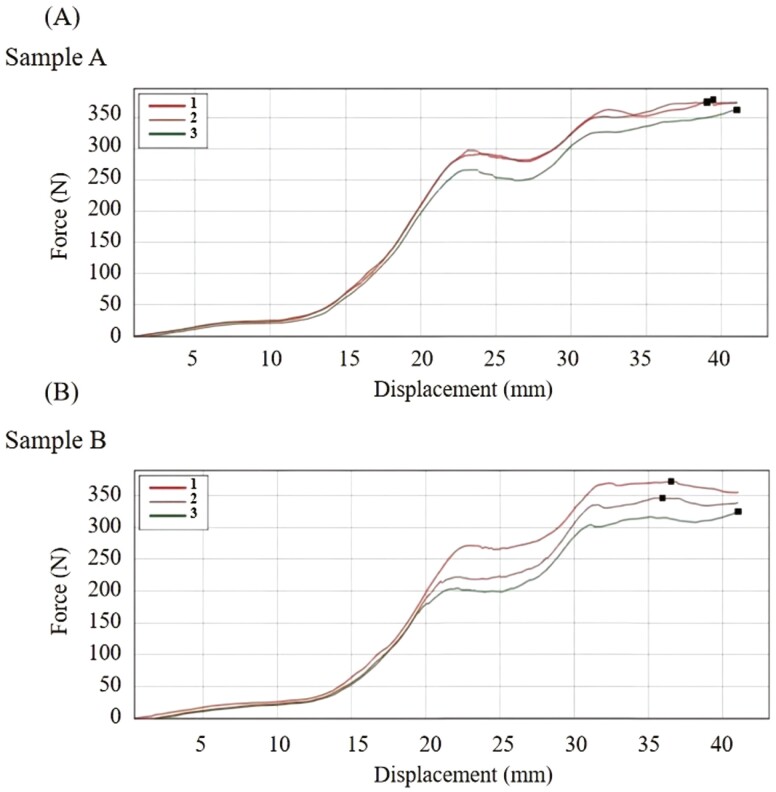
Force–displacement curves for the honeycomb-structured foldable cushion. (A) Force–displacement curves of sample A. (B) Force–displacement curves of sample B. Each curve refers to one repetition of measurement. The enclosed area beneath the force–displacement curve represented the total absorbed energy during displacement.

The force–displacement curves of the honeycomb-structured foldable cushion under bi-axial compression are shown in Figure 4. Two honeycomb-structured foldable cushion samples (i.e., sample A and sample B) were tested, with each of these two samples subjected to three repetitions. The summary of the energy absorption performance indices, including the peak supporting force and total absorbed energy is provided in Supplementary Table 3. During each test on both samples, the supporting force exhibited a rapid peak at approximately 40 mm of deformation length, followed by a sustained plateau phrase.

### Protection Rate

Two complementary fall tests, namely the touchdown fall test and the non-touchdown fall simulation test, were conducted, involving a total of 21 human subjects (10 males and 11 females). The touchdown fall test comprised 14 young and middle-aged adults, aged 18 to 40 (mean ± *SD*: 27.6 ± 8.8) years, whereas the non-touchdown fall simulation test involved seven older subjects, aged 62 to 73 (mean ± *SD*: 65 ± 3.7; Table 1) years. The mean BMI ± *SD* was 22.6 ± 2.0 kg/m^2^ for individuals in the touchdown fall test group and 23.0 ± 1.2 kg/m^2^ for those in the non-touchdown fall simulation test group. A total of 603 touchdown fall tests were performed, with minimum, maximum, and average test numbers of 16, 77, and 43.1 per subject, respectively. A total of 254 non-touchdown fall simulation tests were carried out, with minimum, maximum, and average test numbers of 23, 70, and 36.3 per subject, respectively. In the touchdown fall test, the IBPV demonstrated a mean protection rate of 84.3%, ranging from 72.7% to 93.8% for each individual. The mean protection rate for the non-touchdown fall simulation test was 84.7%, ranging from 82.6% to 88.0%. Furthermore, the mean failed protection rate and invalid protection rate of the IBPV for the touchdown fall tests were 4.9% and 2.3%, respectively, whereas for the non-touchdown fall simulation tests, these rates were 4.7% and 2.1% (Table 1).

**Table 1.** Basic Characteristics of Study Subjects and Protection Rate of the Intelligent Bone Protection Vest

Subjects no.	Age (years)	Sex	Body weight (kg)	Height (cm)	BMI (kg/m^2^)	Total falls (*n*)	Successful protection (*n*)	Failed protection (*n*)	Invalid protection (*n*)	Protection rate (%)^*^
Touchdown fall test										
1	20	female	54	158	21.6	77	56	8	13	72.7
2	21	male	69	172	23.3	72	55	9	8	76.4
3	19	male	67.5	165	25.0	65	58	2	5	89.2
4	20	male	68	168	24.1	53	44	7	2	83.0
5	22	female	55	155	22.9	51	45	4	2	88.2
6	35	male	60	165	22.0	43	37	6	0	86.1
7	40	female	55	160	21.5	42	37	5	0	88.1
8	22	female	57.5	160	22.7	41	36	3	2	87.8
9	23	female	50	162	19.1	41	38	3	0	92.7
10	27	male	55	170	19.0	32	26	6	0	81.3
11	36	female	56	160	21.9	28	23	5	0	82.1
12	48	female	57.5	160	22.7	26	20	6	0	76.9
13	21	male	70	170	24.2	16	15	1	0	93.8
14	33	male	75	170	26.0	16	13	3	0	81.3
Average	27.6	NA	60.7	163.9	22.6	43.1	35.9	4.9	2.3	84.3
Non-touchdown fall simulation test										
1	62	female	55	155	22.9	23	19	4	0	82.6
2	62	female	55	160	21.5	31	27	4	3	87.1
3	63	female	54	158	21.6	46	38	8	0	82.6
4	65	male	68	168	24.1	70	53	11	6	82.8
5	73	male	68	165	25.0	28	24	2	2	85.7
6	67	male	69	172	23.3	25	22	1	2	88.0
7	63	female	58	160	22.7	31	26	3	2	83.9
Average	65	NA	61	162.6	23.0	36.3	29.9	4.7	2.1	84.7

*Notes*: BMI = body mass index.

^*^The percentage of successful protection among total falls.

### User Experience

Fourteen interviewees were included in the semistructured interviews. Six main themes emerged from the interviews, including (i) protective effects, (ii) effectiveness of protection, (iii) usability, (iv) pre- and post-fall feelings, (v) basic characteristics, and (vi) cost-effectiveness. Each theme and its respective subtheme are described below and illustrated by participant quotes (Table 2).

**Table 2.** Summary of Themes and Subthemes

Themes and subthemes	*n* (%)
1. Protective effects	11 (78.6)
1.1 lumbar support and protection	
1.2 Cushioning effect	
2. Effectiveness of protection	12 (85.7)
2.1 Adequate coverage of critical body parts	
2.2 Prompt deployment	
2.3 High protection rate	
3. Usability	9 (62.3)
3.1 Inapplicable scenarios	
3.2 Usage scenarios	
4. Pre- and post-fall feelings	10 (71.4)
4.1 Comfort	
4.2 Softness	
4.3 Without painful feeling	
5. Basic characteristics	11 (78.6)
5.1 Acceptable weight for wearing	
5.2 Belt size diversity	
5.3 Non-obtrusive exterior design	
6. Cost-effectiveness	2 (14.3)
6.1 Acceptable price	
6.2 Reusability	

The first theme, mentioned by most participants (*n* = 11, 78.6%), centered around the cushioning effects and lumbar support provided by the IBPV. Participants recognized the device’s capacity to safeguard against harm and provide lumbar support, as well as its ability to cushion impacts, thereby mitigating hip injuries resulting from falls. However, it is noteworthy that one participant noted challenges in perceiving the protective effects of the device due to her hunchbacked condition.

*The cushioning carried my weight. Even if I fall hard, I feel no pain or risk of injury. (Female, 60 years old)*

*The belt has just the right width for back support and can provide good lumbar protection. (Male, 63 years old)*

*Since I have a severely curved back, I slightly felt unprotected when sitting down, as well as an upward shift of the device. (Female, 76 years old)*

Twelve out of 14 (85.7%) participants emphasized the protection efficacy of this wearable device. The deployment of the honeycomb-structured foldable cushion, offering comprehensive coverage of vital body areas including the hips and thighs, contributed to the device’s effectiveness in providing protection. Moreover, participants in the non-touchdown fall simulation test recognized its high protection rate across a range of simulated real-life scenarios.

*When a fall occurred, the device deployed quickly, which is very good. (Male, 63 years old)*

*When I fell, the foldable cushion opened fast. It provided great cushioning and protected my hips and mid-thigh. (Male, 69 years old)*

Over 60% of the participants mentioned the usability of this wearable device. This device was regarded as effective in protecting against fall-induced injuries due to its ability to deploy the honeycomb-structured foldable cushion in various real-life scenarios. In addition to its protective features, demonstrated versatility by serving as a small stool to provide hip support when wearers experienced fatigue during walking. Nevertheless, certain concerns regarding the device’s usability were raised, with one participant noting that it might be inconvenient and uncomfortable to wear in environments such as the bath or shower, where many falls tend to occur.

*Deploy this foldable cushion when your legs are aching or sore. It can serve as a little stool for a while. The cushion is very soft. (Female, 63 years old)*

*It would be inconvenient to use the shower with this device worn. (Female, 61 years old)*

Ten out of 14 (71.4%) participants described the feelings of wearing this device both before and after conducting the fall tests. In the context of effective fall protection, the soft cushion deployed by the device fully encased critical body areas vulnerable to fall-induced injuries, such as the hips and thighs, ensuring that wearers did not experience discomfort or pain. In addition, the lumbar support offered by the belt was commended for its effectiveness in alleviating lower back discomfort.

*The belt supported my lumbar area. It’s pretty comfortable to wear this device for a long time. (Female, 63 years old)*

*Before using it, I worried that there would be discomfort or even a stinging sensation. However, after the cushion deployed, it fit nicely on my hip; I didn’t feel any pain. (Female, 60 years old)*

Another theme, mentioned by most participants (*n* = 11, 78.6%), pertained to the basic attributes of the wearable device, such as weight, size, and exterior design. Overall, participants found the device’s exterior design and weight to be well-suited to their needs. Although one participant noted that the device felt slightly heavy when held, this concern was mitigated when the device was worn. Suggestions for improvement were raised, including the availability of a wider range of belt sizes.

*Holding the device in the hand was a bit heavy; however, after putting it on, the weight was dispersed and acceptable. (Female, 60 years old)*

*The design of this product is not obtrusive, and the belt width is suitable. (Male, 82 years old)*

*I’m too skinny. There should be more belt sizes to choose from. (Male, 69 years old)*

Two participants commented on the cost-effectiveness of the IBPV, noting that its reusability and reasonable pricing rendered the device a cost-effective choice.

*This product suits me, the price is not too high and acceptable. (Male, 79 years old)*

*The cushion can be folded after deployment, which is critical. The price is acceptable. (Male, 69 years old)*

## Discussion and Implications

Population is aging at an unprecedented pace globally. It is imperative to identify and optimize advanced technologies aimed at enhancing the protection of older adults against the risk of falls and fall-induced injuries. We conducted a comprehensive evaluation of the mechanical properties, effectiveness, safety, and user experience of the IBPV—an innovative, reusable, non-airbag wearable device. The compression experiments demonstrated the energy absorption capacity of the honeycomb-structured foldable cushion. Results from the fall tests showed that the IBPV provided an average protection rate of 84.3% and 84.7% among young and middle-aged subjects and older subjects, respectively. These findings highlight the potential of the IBPV in reducing the incidence of fall-induced injuries by mitigating the impact force on the hip resulting from falls. In addition, user feedback, acquired via semistructured interviews, yielded supplementary insights into the advantages of the IBPV concerning its protective efficacy, usability, fundamental attributes, cost-effectiveness, and the experiences of wearers both before and after falls. The IBPV’s potential areas for improvement were also pinpointed, specifically the requirement to expand the variety of available sizes and styles.

Compared to mainstream active wearables that utilize airbag technology, the IBPV enhances the active fall protection technology through three pivotal enhancements. First, its honeycomb-structured foldable cushion has an intriguing feature for easily folding and resetting after deployment, permitting the device’s reusability. This feature is critical for older adults with mobility challenges who require long-term care, particularly those suffering from neurodegenerative disorders, such as Parkinson’s disease and Alzheimer’s disease (Allen et al., 2010; Ansai et al., 2017; Borges et al., 2015). Second, in addition to mitigating the hip impact forces, this wearable device provides lumbar support, potentially alleviating lower back discomfort and pain. Third, its reusability and relatively affordable price contribute to its favorable cost-effectiveness, rendering it a practical option for widespread adoption among older adults. Lastly, upon deployment and placement over the hip, the honeycomb-structured foldable cushion promptly offers initial peak supporting force, stabilizing during compression. Wearers might perceive a sensation akin to sinking into the mud, elucidating the perceived softness of the foldable cushion upon impact. This compressive mechanical characteristic distinguishes it from the airbag, which functions more as a spring due to its strong rebound effect under compression (Tanaka et al., 2009).

We acknowledged several limitations of the IPBV and current pilot study. First, similar to active wearables using airbag technology, this device is unsuitable for use during bathing or showering, where many falls occur (Tarbert et al., 2023). Second, the device’s efficacy is highly contingent on the precision of the machine learning-based algorithm responsible for real-time fall detection. In the pilot studies, notably for some participants (e.g., subjects 1 and 2) involved in the touchdown fall tests, the number of invalid protection cases was relatively high, which lowered the protection rate. This indicates that additional improvements to enhance algorithm accuracy are needed. Third, although the compression tests provided valuable insights, we recognize the need for impact tests (e.g., drop-weight impact test) to fully assess the resistance and energy absorption capabilities of the honeycomb-structured cushion. This raises potential concerns about the extent of the IBPV’s protective effects. Future research should focus on filling this critical gap regarding the mechanical properties of this innovative material. Fourth, the touchdown fall test conducted was designed to mimic natural falls in various real-life scenarios. Despite multiple replications of these fall simulations for each scenario, they may not accurately replicate natural falls in terms of direction, speed, or magnitude. Fifth, due to safety considerations, the majority of participants involved in the fall test were young and middle-aged; however, the primary intended users of this device are older adults. The BMI ± *SD* values for the touchdown and non-touchdown fall tests were 22.6 ± 2.0 and 23.0 ± 1.2 kg/m², respectively, indicating our participants were mainly of healthy weight and not representative of obese individuals. Therefore, there is a necessity for a more extensive evaluation within a larger and representative population requiring such a protective device. Moreover, although we qualitatively evaluated the usability, safety, and opportunities for further optimization of the IBPV, the data collected were based on the wearer’s immediate experience, potentially overlooking the challenges of long-term compliance. Lastly, the device’s size and style options are presently limited, possibly necessitating better-fitting designs to accommodate individuals with extremely small or large waist circumferences. It is noteworthy, however, that additional styles, such as belt-style and shoulder strap-style, have been developed and manufactured since the time of this report.

In summary, our study demonstrated that the IBPV—a novel, reusable, non-airbag wearable device—exhibited ability in preventing fall-induced injuries. The compression experiments elucidated the energy absorption capabilities of the honeycomb-structured foldable cushion integrated within the IBPV. Following a series of over 800 cumulative fall tests involving 14 young and middle-aged participants and seven older subjects, the IBPV exhibited an overall protection rate exceeding 84%. Moreover, the user experience analysis further provided valuable insights into the strengths of the IBPV, as well as areas in need of improvement. Future studies with more rigorous methodologies, such as comprehensive hip impact force measurements, larger sample sizes, and extended follow-up periods, are warranted to confirm whether this active wearable device may serve as a dependable fall protection product.

## Supplementary Material

igae066_suppl_Supplementary_Tables

## Data Availability

Data and material assessed in this study can be requested by contacting the corresponding author. The study reported in the manuscript was not preregistered.
