# Supplementary material for: Mechanical Property, Efficacy, and User Experience of an Innovative Wearable Device in Preventing Fall-Induced Injuries
Source: Innov Aging. 2024 Jul 12;8(8):igae066. doi: 10.1093/geroni/igae066 (PMC11310589; doi:10.1093/geroni/igae066)
Supplement: igae066_suppl_Supplementary_Tables [file igae066_suppl_supplementary_tables.docx]

Innovation in Aging Supplementary Material: Hu et al. Mechanical Property, Efficacy, and User Experience of An Innovative Wearable Device in Preventing Fall-Induced Injuries.

**Supplementary Table 1.** Summary of the energy absorption performance of the honeycomb-structured foldable cushion

| Sample | Peak supporting force (N) | Total absorbed energy (mJ) |
| --- | --- | --- |
| A |  |  |
| Test 1 | 377.6 | 6988.4 |
| Test 2 | 374.0 | 6966.4 |
| Test 3 | 361.8 | 6755.2 |
| B |  |  |
| Test 1 | 371.2 | 6676.3 |
| Test 2 | 346.0 | 6436.8 |
| Test 3 | 324.1 | 6033.8 |

**Supplementary Table 2.** Questions asked during the semi-structured interview

| 1. What are your thoughts on this device, particularly regarding its weight, size, and other characteristics? |
| --- |
| 1. How did you feel when wearing this device before you fell? |
| 1. How did you feel when you fell down while wearing this device? |
| 1. How do you think this product could be improved? |
| 1. Is there anything about this product that you are particularly happy or unhappy with? |

**Supplementary Table 3.** Deployment time consumption tests results of the honeycomb-structured foldable cushion

| Test No. | Start time (s) | End time (s) | Time consumption (s) |
| --- | --- | --- | --- |
| Sample A |  |  |  |
| 1 | 4.67 | 4.89 | 0.22 |
| 2 | 7.20 | 7.43 | 0.23 |
| 3 | 1.21 | 1.44 | 0.23 |
| 4 | 0.15 | 0.34 | 0.19 |
| 5 | 0.89 | 1.10 | 0.21 |
| Sample B |  |  |  |
| 1 | 0.26 | 0.49 | 0.23 |
| 2 | 15.97 | 16.18 | 0.21 |
| 3 | 8.31 | 8.50 | 0.19 |
| 4 | 2.92 | 3.13 | 0.21 |
| 5 | 9.55 | 9.78 | 0.23 |
| Average | NA | NA | 0.21 |
